# Positive Emotions, Spirituality and the Practice of Psychiatry

**DOI:** 10.4103/0973-1229.36504

**Published:** 2008

**Authors:** George E. Vaillant

**Affiliations:** * Professor of Psychiatry, Harvard Medical School, USA

**Keywords:** *Neurobiology*, *Positive emotions*, *Religion*, *Spirituality*

## Abstract

This paper proposes that eight positive emotions: awe, love (attachment), trust (faith), compassion, gratitude, forgiveness, joy and hope constitute what we mean by spirituality. These emotions have been grossly ignored by psychiatry. The two sciences that I shall employ to demonstrate this definition of spirituality will be ethology and neuroscience. They are both very new. I will argue that spirituality is not about ideas, sacred texts and theology; rather, spirituality is all about emotion and social connection.

Specific religions, for all their limitations, are often the portal through which positive emotions are brought into conscious attention. Neither Freud nor psychiatric textbooks ever mention emotions like joy and gratitude. Hymns and psalms give these emotions pride of place. Our whole concept of psychotherapy might change if clinicians set about enhancing positive emotions rather than focusing only on negative emotions.

He is forever free who has broken
Out of the ego-cage of I and Mine
To be united with the Lord of Love.
This is the supreme state. Attain thou this
And pass from death to immortality

**—Bhagavad-Gita, Chapter 2, trans. Mahatma Gandhi (Fowler, 1984)**

## I. Introduction

As the 21st century begins, a great many people - especially in the English-speaking world - are in search of some kind of common spiritual ground. On the one hand, science, increasing education and intolerance for patriarchal dogma has led to a steady erosion of the membership in mainstream churches and temples. On the other hand, this shift toward secularism has created an equally steady increase in evangelical, Pentecostal and unabashedly emotional New Age religions. Many of the best sellers of the last five years in America have been based either on the popular search for the spiritual or the rejection of the religious in a secular world.

In an effort to scientifically validate this thirst for spirituality, I wish to build on the relatively new scientific disciplines of cultural anthropology, ethology (animal behaviour) and neuroscience - all of which study the so-called positive emotions such as awe, love, joy, hope, faith/trust, forgiveness, gratitude and compassion. Positive emotions, *per se*, are present in most mammals and have been experimentally shown to help humans behave communally and to learn more quickly (Lyubomirsky *et al.*, 2005). Surprisingly, spirituality is virtually indistinguishable from these positive emotions and is, thus, rooted in our evolutionary biology. Because these emotions are also the same ones for which most religions strive, spirituality is a common denominator for all faiths.

### Positive Emotions Make Religion Nourishing

In emphasizing the power of emotions like love, compassion and forgiveness, I wish to perform for spirituality what the science of nutrition performed for the world's discordant diets. Just as nutrition identified the vitamins and the four basic food groups and made other peoples’ “disgusting” ethnic diets appear nourishing, similarly, by focusing on neuroscience and ethology, I will suggest that it is positive emotions that make religions nourishing, not their warring cognitive ideologies.

Recently, I tentatively began to discuss spirituality with a close friend of mine, a brilliant woman and a devout Anglican. “When I hear the word spirituality,” she exploded, “I break out in spots!” I was surprised to hear her voice her sentiment so strongly; but to her, spirituality was no more than an illusion. The problem, of course, is that the word “spirituality” has many meanings. While spirituality is both the source and the outgrowth of faith for many people, for just as many others, it is considered suspect. For the devoutly religious (among others), “spirituality” can be equated with the occult and with bogus faith healers; it often brings to mind reincarnation, telepathy, crystals, angels and Tarot cards. To others, less burdened with a belief system, spirituality can appear as nothing more than covert narcissism and a New Age mandate to follow your bliss. I believe these mindsets to be terribly wrong.

### Spirituality, Positive Emotions and Psychiatry

When I use the term spirituality, I am suggesting that spirituality is all about positive emotions. These emotions include *love, hope, joy, forgiveness, compassion, trust, gratitude* and *awe*. Of enormous importance is the fact that none of the eight are “all about me.” They epitomize what Charles Darwin called social emotions; they all help us “to break out of the ego cage of I and mine.”

So why does psychiatry know so little about the positive emotions? Negative emotions such as fear, anxiety, sadness and anger are also inborn, and psychiatry knows a whole lot about them. The problem is that negative emotions are all dedicated to individual survival. Negative emotions are “all about me.” In contrast, positive emotions free the self from the self. We feel the emotions of both vengeance and of forgiveness deeply, but the long-term results of these two emotions are very different.

Negative emotions are often crucial for survival – but only in time present. The positive emotions are more expansive and help us to broaden and build (Fredrickson, 2001). They help us to survive in time future. Careful experiments have documented that while negative emotions narrow attention and miss the forest for the trees (Fredrickson, 2004), positive emotions, especially joy, make thought patterns more flexible, creative, integrative and efficient (Isen *et al.*, 1991; Panskepp, 1998; Lyubormirsky *et al.*, 2005). When we are frightened, angry or depressed, it is hard to be creative or to learn new things.

Not only are the sciences that have made positive emotions tangible to academics very new, but also emotion has often been an unwelcome guest at the academic table. Until the last twenty years, however, we knew very little about the positive emotions. We may mumble words like hope, love and joy, but then we roll our eyes or change the subject. In their lengthy and influential textbooks that laid the foundations for scientific psychology, Wilhelm Wundt (1896) and William James (1893) each allotted a single chapter - and a somewhat disdainful one at that - to the emotions. A half-century later, Burrhus F. Skinner (1953), a brilliant and most rational psychologist, dismissively proclaimed, “The ‘emotions’ are excellent examples of the fictional causes to which we commonly attribute behavior.” Try telling that to a neuroscientist like Antonio Damasio in 2007.

More astonishing, however, is that the discussion of positive emotions is even more distasteful than a discussion of negative emotions. Freud was good on negative emotion, but he tried to keep music (often a surrogate for positive emotion) out of his house. Consider the fact that the leading American text, *The Comprehensive Textbook of Psychiatry*, half a million lines in length, devotes 100–600 lines each to shame, guilt, terror, anger, hate and sin and, of course, devotes thousands of lines to depression and fear/anxiety. In contrast, the textbook devotes only five lines to hope, one line to joy, and not a single line to faith, compassion, forgiveness or love (Sadock and Sadock, 2005). What is there about these words that make us afraid to discuss them? I don't know. But I do know that the word “joy” - never mentioned by Freud - is mentioned over and over again in hymnals from all denominations. The psalms give gratitude and compassion pride of place. In short, the cultural memes of religion may kill thousands, but the silver lining is that religions offer positive emotions a safe portal to enter into the consciousness of billions. In psychoanalysis we sometimes dismiss positive emotion as “a flight into health,” and we call love “object relations”!

### Survival Value of Unselfish Positive Emotions

My emphasis on positive emotion (aka spirituality) does not mean that psychiatry should ignore the more selfish, if equally valuable, negative emotions. Nor do I wish to deny that many genes are “selfish.” I merely wish to assert the survival value conferred by unselfish positive emotions (aka spirituality) in the long run. True, “evil” probably occurs today at the same per capita rate as it did in the Iron Age - before Buddhism and the wisdom of the Bhagavad-Gita brought deep compassion to Hinduism, and before the New Testament softened the earlier Judaic law of an eye for an eye and a tooth for a tooth. I only ask that psychiatry also pay attention to the positive emotions (Vaillant, in press).

For example, for most of the 20^th^ century virtually all psychologists ignored the positive emotion of love - if love is defined as “attachment” rather than “lust.” In his 1958 presidential address to the American Psychological Association, Harry Harlow (1958, p. 673) was driven to exclaim, “Psychologists, at least psychologists who write textbooks, not only show no interest in the origin and development of love or affection, but they seem to be unaware of its very existence.” John Bowlby (1979), a mid-century psychoanalyst and an ethologist who stressed the importance of positive attachment, was largely ignored by the psychiatric establishment for the first decade after his groundbreaking work.

A Darwinian like Richard Dawkins (1976) might wonder how the principle of unselfish love could be a hardwired and useful evolutionary trait. But consider the following surprising facts: the average longevity of major American companies is less than forty years; great personal fortunes rarely survive more than three generations; few nations, and still fewer dynasties, have lasted more than 300 years. In contrast, the world's great religions - Buddhism, Hinduism, Christianity, Islam and Judaism - share two things in common: they are all based, in part, upon a commitment to unselfish love and they have all endured for more than 1400 years. The Benedictine order, founded on the overarching principle of “take care of the sick,” has lasted 100 times longer than the Nazi order which was founded on the seemingly sound evolutionary principle of “might makes right” and that only the fit should survive. It is not a miracle that the Benedictines have lasted longer than the Nazis. It is how human beings are wired for social survival. For the last million years, survival by our ancestors in the African savanna would have been impossible without the natural selection for prosocial emotions.

On those sparsely wooded plains evolved our hairless ancestors. They lived in a land richly endowed with carnivores. Our ancestors could not run like the gazelle, burrow like the rabbit, climb trees like the gibbon, fly away like the flamingo, or fight back like the elephant. If they did not band together, they perished. They did not even have fur (like apes do) for their young to cling to; instead, the mother had to cling to her young. In order to survive humans had to sometimes subordinate both hunger and sex for the development of an inborn altruistic social organization. From such social bonding came lasting attachment and the survival of their young.

The evolution of organized religion accompanied the evolution of stable settlements seven to twelve millennia ago. But for the survival of cities, cultural as well as genetic evolution was needed. Until the transformative millennium extending from 600 B.C. to 700 A.D., the world's great cities emerged only to disappear. Ur, Babylon, Mohenjo Daro, Carthage, Machu Picchu, the Mayan metropolis of Tikal, and the great early Chinese and Egyptian capitals have all vanished beneath sand, fields and jungle creepers. Not until Hinduism shifted away from animal sacrifices for appeasing external deities to moving moral responsibility inside; not until Buddhism, Christianity and Islam put mercy ahead of righteousness and asceticism; not until organized religions emerged that emphasized the positive emotions of love and compassion rather than fear and dominance; not until then did great cities endure. If genetic evolution of the primate limbic system allowed, through natural selection, the development of the “empathic” mirror cells and the unselfishly loving spindle cells that allowed our hunter-gatherer ancestors to keep their children safe from predators and hunger on the African savannas, so also did cultural evolution facilitate the emergence of compassionate religions that allowed once xenophobic tribes to live cooperatively together in urban cities and in the two millennia old Roman and Chinese empires.

## II. Spirituality, Religion and the Limbic System

Spirituality and religion differ from each other in important ways. The term “religion” usually refers to the *interpersonal and institutional* aspects of religio-spirituality that are derived from engaging with a formal religious group's doctrines, values and traditions. However, when I use the term “spirituality” I am referring to the *psychological experiences* of religio-spirituality that relate to an individual's sense of connection with a transcendent power (be it a single deity or anything else considered to be “larger” than one's self) with feelings of awe, gratitude, compassion and forgiveness. Spirituality and religion are both born in our brain, but there are important differences. First, religion draws circles that draw others out, whereas spirituality draws circles that draw others in. Second, spirituality is based on our biology, whereas religion is based on our culture. If this generalization is true, ethnic and national groups should differ in terms of their religious beliefs but resemble each other in their spiritual values.

Let me illustrate this second difference. If I were to ask an international congress of psychiatrists to nominate the six greatest religious exemplars of the 20^th^ century, there would be no agreement. If I were to ask the same congress to name the six greatest spiritual exemplars of the 20^th^ century, three men - Nelson Mandela, Mohandas Gandhi and Martin Luther King, Jr. - would be on almost everybody's list. However, please note there would have been no such agreement had I asked the audience to use “left-brain” words rather than “right-brain” images to define spirituality.

Let me try to explain further our lack of agreement on religion and our relative agreement on spirituality. Just for a moment imagine the human brain divided into three parts. At the center is the reptilian brain. It has evolved relatively little in the last 100 million years and contains the selfish and survivalist primitive emotions and the hypothalamic instincts that serve both scientists and crocodiles equally well (for feeding, fighting, fleeing and fornicating).

Surrounding the primitive brain is the limbic brain. In 1878, the French anatomist and father of neurosurgery, Paul Broca (1878), announced his findings that the brains of all mammals contain a structure absent from the brains of reptiles - the limbic system. Only since I graduated from medical school, however, has neuropsychiatry come to regard the limbic system as being as important as the hypothalamus (LeDoux, 1998; MacLean, 1990; Panksepp, 1998). Only in the last 20 years has the anterior cingulate gyrus, and only in the last 10 years has the insula, been seen as being equal in importance to the hippocampus and the amygdala. Primate altruistic attachment (Harlow, 1958; Bowlby, 1979; Zubietta, 2003) and primate empathy via spindle cells (Allman *et al.*, 2001, 2005) and via mirror cells (Carr *et al.*, 2003; Rizzolati, 2005) are all very recent discoveries. The first article specifically on “the neurobiology of the positive emotions” (Burgdorf and Panksepp, 2006) has only been published in the last year.

Although neuroanatomists differ as to the limbic structures that should or should not be included in our definition of the limbic system, a majority of investigators would include the hippocampus, the amygdala, the insula, and the anterior cingulate and the ventromedial prefrontal cortices. For the last 100 million years the limbic system evolved steadily in our mammalian ancestors but not in crocodiles. Dinosaurs, after all, survived for 100 million years (until their extinction 65 million years ago) without their brains getting any bigger at all. Only in mammals, and especially in primates, has natural selection campaigned for a more complex brain instead of bigger teeth, brighter colours or a thicker hide. It is the mammalian limbic system that plays the greatest role in positive emotions; it is the *Homo sapiens*′ limbic system that plays the greatest role in spirituality (Hamer, 2004).

Remove a mother hamster's entire neocortex and she will seem feeble-minded in a psychologist's maze, but she can still unselfishly love and raise her pup. Damage her limbic system only slightly and she can still be a wizard at mazes, but will be an utterly incompetent mother (Panksepp, 1998). Love lives within the limbic medial temporal lobe - where smells, music, caretaking and memory all come together - and most especially in the limbic system's anterior cingulate gyrus (Panksepp, 1998). The unifying affect of joy, of awe, of the transcendent appears to arise from limbic structures (Panksepp, 1998).

### Cognitive Religion and Emotional Spirituality

Covering the limbic system like a helmet is the neocortex that in our human ancestors (but not in golden retrievers) has evolved most spectacularly - more than doubled its weight - over the last two million years. The divisive cognitions of language, culture, ideology, and religious dogma spring primarily from our neocortex. Many dogs that are raised in kind and loving families radiate limbic faith, hope, forgiveness and love; while some very brilliant scientists and theologians do not. Of course, philosophy, nuclear physics, and theological prose of every shade and stripe live in our *Homo sapiens* neocortex. But when a beloved woman is weeping, both golden retrievers and two-year-old children will rush thoughtlessly, speechlessly, unselfishly - spiritually - to her side to provide comfort. Consider how a kitten's or a human infant's limbic separation cry evokes an unselfish response in almost all of us. In short, I am suggesting that we are better at identifying spiritual exemplars than religious ones because different areas of our brains are involved in making the decision. Neocortical cognitive religions divide, whereas limbic emotional spirituality unifies.

The effect of positive emotion on the autonomic nervous system has much in common with the relaxation response to meditation that was popularized by a Harvard professor of medicine, Herbert Benson (1996). Indeed, positive emotion and spiritual experience cannot be disentangled. Benson (1996) reported that 80% of his meditators chose a religious symbol as a mantra for meditation. Another laboratory noted that 45% of people sensed the sacred during meditation and that 68% experience a sense of the sacred after childbirth (Kantrowitz *et al*., 1994). In contrast to the fight-or-flight response of negative emotion, which activates the sympathetic nervous system, positive emotion activates the parasympathetic nervous system. Like in meditation, positive emotions - such as joy, compassion, attachment, trust and forgiveness - lower metabolism, blood pressure, heart rate, respiratory rate and muscle tension. Indeed, if sleep slowly lowers our basal metabolism by 8%, meditative states lower our metabolism 10 to 17% (Benson, 1996).

### Right-Left Prefrontal Activity

Richard Davidson is a University of Wisconsin neuropsychologist who has built his distinguished career on studies clarifying that in people with gloomy introverted personalities, the right prefrontal brain (above your right eye socket) is biologically more active than the left prefrontal brain. In people with sunny outgoing personalities, the left prefrontal brain is more active than the right. Studying the brain activity of a devout Tibetan monk with decades of loving-kindness meditation behind him, Davidson found that the left prefrontal brain activity was higher than in any of 175 normative Westerners that he had tested (Davidson and Harrington, 2002, p.17). My intent here is not, like Freud, to suggest that religious ideation is an illusion but, rather, to suggest that mammalian evolution has hardwired the human brain for subjective spiritual/religious emotional experience.

In our lives, religious beliefs, scientific truths and spiritual emotions all play important roles. For selfish reptiles to evolve into loving mammals it took the *genetic* evolution of our emotional limbic system. For loving, playful passionate mammals to become reflective, cause-seeking, mythmaking *Homo sapiens* it took the *genetic* evolution of our huge human neocortex. Finally, for *Homo sapiens*′ cause-seeking to evolve into 20^th^ century science, and for mythmaking to evolve into religion and then into hospitals and finally the World Health Organization (i.e., the institutionalization of unselfish love), took the far more rapid cultural evolution which, often facilitated by religion, leads to cooperation between the positive emotions of our limbic system *and* the cognitive reason of our neocortex.

From 1960 and 1990, sociobiologists and evolutionary psychologists like Richard Dawkins and Steven Jay Gould regarded the concept of positive group evolution as the worst sort of heresy. Only “selfish” genes were credible. In 1994, Antonio Damasio (1994, p.126) broke ranks when he wrote, “Suprainstinctual survival strategies generate something probably unique to humans: a moral point of view that, on occasion, can transcend the interest of the immediate group and even the species.” More recently, evolutionary biologists (Wilson, 2002; and Silk, 2003) have also suggested that a change in the scientific attitude towards group selection is in order. Evolutionary psychologist Mark Hauser (2006) has also documented that in the evolution of *Homo sapiens* “maturity” and empathy have been positively selected for (Hauser, 2006). Moll and coworkers (2006) have identified limbic regions that, rather than suppressing “baser urges,” find altruistic giving to strangers pleasurable in its own right.

### Examples of Cultural Compassion, and the Role of Religion

The United Nations, the Olympic Games, the Nobel Peace Prizes and the world's response to the 2004 tsunami reflect cultural evolution over the past century. In this same vein, it is only in the last century that humans have learned to treat one another as equals - giving women the right to vote, to be President, to be ordained, and the right to choose or not choose motherhood. But these cultural steps had already been anticipated and were facilitated by a brain hardwired to feel pleasure at communal efforts and compassion towards strangers in distress.

Over the last century, with empires disappearing and state religions hobbled, Europe has - with ever-evolving unanimity - agreed that Africa belongs to the Africans and that Europe needs to ask for African forgiveness for four centuries of colonial exploitation. With every African famine and epidemic the world has responded with increasing, if imperfect, compassion. I believe that the difference has been made by cultural evolution. Just as the genetically-derived limbic system, with its positive emotions, facilitated the survival of mammals over dinosaurs, so did evolving cultural focus on positive emotions contribute to the communal survival and the success of *Homo sapiens*. After all, since the beginning of recorded history, 60 years (from 1945 to 2005) is the longest period of time over which one European nation has failed to declare war on another.

Religion, of course, has played a very uneven role in this cultural evolution. On the one hand, religious beliefs have provided cultural justification for some of the most heinous human crimes ever committed. On the other hand, for all their intolerant dogma, religions have provided communities a unifying view of the human condition and have often provided the portal through which positive emotions are brought to conscious attention. For example, neither Freud nor psychiatric textbooks ever mention emotions like joy and gratitude, but religious hymns and psalms give these emotions pride of place.

As Albert Schweitzer (1947), a deeply religious physician *and* an integrative scientist maintained, “Man can no longer live for himself alone. We realize that all life is valuable, and that we are united to this life. From this knowledge comes our spiritual relationship to the universe.”

## III. Cultural Anthropology, Ethology, Neuroscience and -Positive Emotions

The “scientific” acceptance of positive emotions has been very recent. Our scientific understanding of positive emotions depends on three new sciences, all younger than atomic physics - cultural anthropology, ethology and neuroscience. The neuroscience of the positive emotions came of age with the fMRI, which made such emotions tangible instead of being the allegedly fuzzy notions of unpsychoanalyzed religious devotees. Most of what we know about the neuroscience of positive emotion has been published only in the last 20 years.

Items from Robert Cloninger's (1994) Scale of Self-Transcendence has allowed neuroscientists and geneticists to demonstrate that neural experience of spiritual experience is in part genetic (Hamer, 2004; Eaves, 1999) and that spiritual experience becomes visible with brain-imaging studies (Newberg and D'Aquilli, 2001).

Representative items from the Cloninger scale include the following:

#3 I often feel that I am a part of the spiritual force on which all life depends.

#5 Sometimes I feel so connected to nature that everything seems to be part of one living organism.

#8 Sometimes I feel a spiritual connection to other people that I cannot express in words.

#12 I have had personal experiences in which I felt in contact with a divine and wonderful spiritual power.

#13 I have had moments of great joy in which I suddenly had a clear deep feeling of oneness with all that exists.

These same statements could describe the experiences in deep meditation (Benson, 1996), in childbirth, in temporal lobe epilepsy (Dewhurst and Beard, 1970) and in near-death experiences (van Lommel *et al*., 2001). In part, they are catalyzed by limbic oxytocin and endorphins. By reducing neocortical inhibition, meditation stimulates the limbic system from the outside in (Newberg and Iversen, 2003). Temporal lobe epilepsy and endorphin-driven near-death experiences stimulate the limbic system from the inside out. But Cloninger's spiritual statements are also common reflections of the unselfish attachment between parent and child. They are feelings perhaps subserved by the newly discovered and already mentioned spindle cells and mirror cells that are more highly developed in humans than in primates and are essential for raising offspring that remain helpless for five to ten years or, in the minds at least of their parents, for perhaps 25 years.

Certainly, spirituality is not just about following your bliss. Spirituality has a deep psychobiological basis and a reality that needs to be better understood. I believe that by taking the science of positive emotions seriously, we can make spirituality palatable, even useful, to religions′ critics and, simultaneously, help those enthralled by their own faith traditions to appreciate what they have in common with the faith traditions of others.

## IV. Connection with Psychiatry

But what does this discussion have to do with the practice of psychiatry? If psychiatrists would only pay more attention to the positive emotions they may more effectively help their patients paralyzed by the “all about me” emotions of depression and resentment. Increased faith, hope and love decreases pain and alleviates suffering, whether these positive emotions are facilitated by religious institutions, caring doctors, nurses and teachers, or by sobriety and/or spiritual meditation.

An illustration of the power of positive emotions to heal in Western psychiatry comes from the 1950s. Until the 1954 discovery of chlorpromazine, the world literature uniformly maintained the efficacy of insulin coma in schizophrenia. After the discovery of an effective antipsychotic neuroleptic, investigators became confident and bold enough to risk conducting adequately controlled studies of insulin coma. By excluding all confounders, they demonstrated that the crucial ingredients were the vastly better nursing care the insulin coma patients received, the better morale of their doctors and the social cohesion of the patient treatment groups (Cramand, 1987) rather than any effect of the insulin-induced coma *per se* (Ackner *et al.*, 1957). The world literature reviewed by Koenig *et al.* (2001) asserts that greater religious involvement reduces depressive symptomatology. For example, Ironson and her collaborators (2002) have shown that increased spiritual involvement not only reduced depression in terminally ill AIDS victims, it mediated increases in hopefulness and in helping behaviours and also decreased serum cortisol.

Psychiatrists bemoan their patients′ narcissism, but through exclusive attention to patients′ negative emotions psychiatrists may contribute to their self-absorption. As psychotherapists we encourage our patients to use up our Kleenex and to dwell on their losses and on their rage towards mean parents. We sometimes forget to support their positive emotions of compassion, forgiveness, joy and gratitude. Alcoholics Anonymous Twelfth Step (alcoholics helping each other) groups discourage the “poor-me's” and resentments and instead they direct a newcomer's attention to loving, forgiving and comforting others. This group support allows the miserable to “broaden and build” toward the future in ways that the well loved and optimistic already do.

Cluster B (externalizing) Axis II personality disorders that are often labeled “borderline” in a derogatory fashion are viewed by psychiatrists as ungrateful cripples who need as much of our patience and ambivalent love as they can pay for (Vaillant, 1992). In contrast, in Alcoholics Anonymous, the “pigeon” (often an emotionally unstable newcomer to the fellowship) is viewed gratefully as someone “who comes along just in time to keep their sponsor sober.” Perhaps instead of complaining about our character-disordered, help-rejecting patients, we might set a good example by assuming a more forgiving, dare I say spiritual, stance. Maybe we should express gratitude to “borderlines” for their help in educating us about psychodynamics, for burnishing our academic reputations, for giving us insights into our counter-transference and for helping us pay off our mortgages. Admittedly, in part, I am jesting, but metaphorically I am quite serious. In both spirituality and psychiatry, the positive emotions of faith, hope, love and gratitude are essential ingredients.

## Concluding Remarks

This article suggests that spirituality reflects positive emotion. Spirituality, like positive emotions, is generated by the limbic system and is more about *us* than *me*. We do not have to be taught positive emotions; our brain is hardwired to generate them. Humanity's task is to pay attention to them, for they are the source of our spiritual being and the key to our cultural evolutionary progress. Spirituality reflects humanity's evolutionary press towards connection and community building even more than it reflects humanity's need for solace and revelation. Although positive emotions have been profoundly neglected by the modern social sciences, organized religions, for all their limitations, have helped to bring positive emotions into the ambit of conscious reflection. The Buddhist ideal is that of the *bodhisattva* - one who elects voluntarily to stay in this world and to help others, rather than entering directly into *nirvana*. Nor is spirituality trivial; if one follows the lives of history's great spiritual exemplars, they have always been community builders, not navel gazers.

### Take Home Message

Psychiatry, with good reason, has paid great attention to the dangers of religious belief. Psychiatry would also do well to study the benefits of positive “spiritual” emotions.

## Questions That This Paper Raises

1. What is the difference between religion and spirituality?
2. Why can we agree on spiritual exemplars, but not religious exemplars?
3. What are the differences between emotion and belief?
4. Why does psychiatry ignore the positive emotions?
5. Are our reasons for ignoring positive emotions the same or different from that which made Sigmund Freud and many other psychiatrists ignore religion? Are the reasons contempt? Fear? Or, is there some other reason?
6. Should, as some modern neuroscientists suggest, the concept of the limbic system be abandoned?

## About the Author

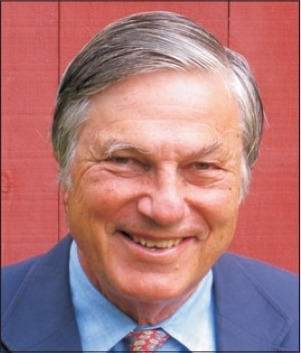



George E. Vaillant is a Professor of Psychiatry at Harvard Medical School and the Department of Psychiatry, Brigham and Women's Hospital. Dr. Vaillant has spent his research career charting adult development and the recovery process of schizophrenia, heroin addiction, alcoholism and personality disorder. He has spent the last 35 years as Director of the Study of Adult Development at the Harvard University Health Service. The study has prospectively charted the lives of 824 men and women for over 60 years. His published works include Adaptation to Life, 1977; The Wisdom of The Ego, 1993; and The Natural History of Alcoholism - Revisited, 1995. His summary of the lives of men and women from adolescence to age 80, called Aging Well, was published by Little, Brown in 2002. His latest book Sprititual Evolution: A Scientific Defense of Faith is in press.

A graduate of Harvard College and Harvard Medical School, Dr. Vaillant did his residency at the Massachusetts Mental Health Center and completed his psychoanalytic training at the Boston Psychoanalytic Institute. He has been a Fellow at the Center for the Advanced Study in the Behavioral Sciences and is a Fellow of the American College of Psychiatrists. He has been an invited speaker and consultant for seminars and workshops throughout the world. A major focus of his work in the past has been on developing ways of studying defense mechanisms empirically. More recently he has been interested in successful aging, positive emotions and spirituality. He is on the steering committee of Positive Psychology. He is also on the Honorary International Editorial Advisory Board of MSM.

Dr. Vaillant has received the Foundations Fund Prize for Research in Psychiatry from the American Psychiatric Association, the Strecker Award from the Institute of Pennsylvania Hospital, the Burlingame Award from The Institute for Living and the Jellinek Award for research in alcoholism. He has twice received research prizes from the International Psychogeriatric Society. Most recently, he received the distinguished service award from the American Psychiatric Association.
